# Interaction of MS prevalence, radon gas concentration, and patient nutrition: a case–control study

**DOI:** 10.1038/s41598-021-96816-4

**Published:** 2021-09-09

**Authors:** Monire Fallah Yakhdani, Mahrokh Jalili, Amin Salehi-Abargouei, Masuod Mirzaei, Abolghasem Rahimdel, Ali Asghar Ebrahimi

**Affiliations:** 1 Department of Environmental Health Engineering, Environmental Science and Technology Research Center, School of Public Health, Shahid Sadoughi University of Medical Sciences, Yazd, Iran; 2 Student Research Committee, Shahid Sadoughi University of Medical Sciences, Yazd, Iran; 3 Nutrition and Food Security Research Center, Department of Nutrition, School of Public Health, Shahid Sadoughi University of Medical Sciences, Yazd, Iran; 4 Department of Epidemiology, Centre for Healthcare Data Modeling, School of Public Health, Shahid Sadoughi University of Medical Sciences, Yazd, Iran; 5 Department of Neurology, Shahid Sadoughi Hospital, Yazd University of Medical Science, Yazd, Iran

**Keywords:** Environmental sciences, Diseases

## Abstract

In general, ecological findings indicate a positive correlation between MS and the intake of certain foods. This study aimed to investigate the relationship between radon (Rn) gas concentration and nutrition of patients in food groups with MS. Demographic information, diet, and building characteristics were collected by a questionnaire. Indoors Rn gas was measured using CR-39 detectors. Three models were used in the study of food intake. The interaction analysis between MS prevalence, diet, residential building characteristics, and Rn gas content was performed using SPSS 2020. The total Rn was significantly associated with cooling devices (*P* = 0.021). Buildings > 20 years had higher Rn concentrations than buildings < 20 years (*P* = 0.038). Also, no significant relationship was found between Rn-total and MS concentrations, but the total Rn concentration was higher in people homes with MS. Case group used more processed meat than the control (*P* < 0.001). The case group consumed more butter than the control, which was significant in Model III (*P* < 0.04). Tomato consumption in the case group was significantly higher than the control (*P* < 0.03). According to the results there was no interaction between Rn gas concentration in any of the food groups in each cases. However, future studies with larger sample sizes will be needed prospectively.

## Introduction

Radon (Rn) is a colorless, odorless, tasteless radioactive gas. The most stable Rn isotope is Rn 222, which is the product of radium 226 decay^1,2^. Radon is widely distributed in the environment, present in varying concentrations in water, soil, sediments, and rocks. Rn concentrations in the atmosphere and outdoors are very low, but in a building, with trapped air, the concentration and consequently its activity level increases rapidly^3^. Therefore, long-term exposure to high concentrations of Rn and its products can cause pathological effects and serious damage to the lungs, causing emphysema, fibrosis, and ultimately lung cancer^4^. Rn breaks down in the lungs and emits alpha particles. The energy of the alpha particle absorbed by the tissues causes tissue death or damage.

Ecological studies have been performed to investigate the association of Rn gas with Multiple sclerosis-MS^5^ in countries with a high prevalence of MS. These countries include the United States^6–8^, Ireland^9,10^, Norway^11^, Sweden^12^, Wales England^13^.

The path of Rn entering closed spaces is such that the Rn in the soil gradually accumulates under the building and its pressure increases. Because the air pressure inside the building is usually lower than the soil, it causes the Rn in the soil to be transferred into the building through the floor and walls and increases its amount. The presence of cracks in the walls, passageways of the building installation network, and any holes in the joints and building materials can be effective in Rn penetrating. The highest amount of Rn is present in the basement and then in the first floors of the building^1^. The limit recommended by the EPA^14^ is 4 pCi/L (148 Bq/m^3^). Precautions should be taken more than this amount and precautionary measures should be taken when the amount reaches 8 pCi/L^15^. The World Health Organization (WHO) has also set a limit of 100 Bq/m^3^ of air for reducing the risks of Rn exposure. If this level can not be observed due to different factors and special conditions of each country, the base r eference level for indoor radon should not be > 300 Bq/m^3^ of air, which is approximately 10 millisieverts per year^16^.

The MS is an autoimmune, inflammatory, and chronic disease of the central nervous system that occurs as damaged nerve or myelin lesions in the white matter of the brain, spinal cord, and optic nerves. This disease is more prevalent among women and at a young age. It often occurs in the age group of 20–40 years. This is diagnosed in advanced stages^17–19^, quite costly and its treatment includes supportive care and symptom management^20^. It is also a chronic debilitating disease with social and economic consequences and has been identified as the second leading cause of disability among young^21^.

Several factors are involved in MS prevalence, including genetic and environmental factors such as nutrition, infection, immunity, viruses, toxins, fuel, and air pollution as nitric oxide, sulfur oxide, carbon monoxide, and PM_10_^22–26^. Unequal geographical distribution of MS (higher prevalence in areas with greater latitude) indicates a significant effect of non-infectious environmental factors such as duration of sun exposure and especially nutritional factors in its occurrence^27^.

Today, a proper diet can play a decisive role in the health of people and is therefore of great importance^28^. In general, the findings of some ecological studies indicate a positive association between MS and the intake of certain foods such as meat, protein, and animal fats^29^. Iran is also geographically classified as a Middle Eastern country, but in terms of MS spread, it has a pattern similar to Western countries^14^. In addition, the results of recent studies indicate the transition of Iranian nutrition over the past two decades and the Iranian dietary pattern change to Western or unhealthy pattern, which is one of the most important underlying causes of chronic MS^30,31^. According to the International Federation of MS, the average prevalence has increased from 30 per 100,000 in 2008 to 33 per 100,000 in 2013. The highest prevalence is in North America (140 per 100,000) and Europe (108 per 100,000) and the lowest in sub-Saharan Africa (1.2 per 100,000) and East Asia (2.2 per 100,000)^32^. According to the WHO classification in the 2008 MS Atlas, Iran is among the regions with a moderate prevalence (20–60 per 100,000 people)^16,33^. Based on the study of the geographical distribution of MS and the incidence of the disease, Iran is one of the regions with a low prevalence^34^.

Yazd is located in the central region of Iran. It has a hot and dry climate with very little rainfall and high dust storms. There are many mines in Yazd province, including uranium. Therefore, this study aimed to investigate the concentration of Rn gas in the air of residential houses of MS and non-MS and its interaction with food groups in Yazd in 2018.

## Materials and methods

The study population consisted of patients with MS^35^ and healthy individuals (control) who were randomly selected from the houses around the patient's residence. Rn gas data and its concentrations were measured using a Rn Gas Monitoring questionnaire in residential buildings^36^ and CR-39 detectors, respectively. The socio-economic status of individuals was measured based on obtained data from the Socio-Economic Status Assessment questionary on variables such as age, marriage, education, ownership status, smoking, medical history, physical activity, stress levels, and sun exposure rate^37,38^. Nutritional data were collected using a 178-item semi-quantitative food frequency questionnaire^39,40^.

### Inclusion criteria

To determine the number of samples, the study was conducted as a pilot study, based on which, referring to the MS Patients Association, patients were contacted. People who were willing to cooperate filled out a form of informed consent. Inclusion criteria for case subjects included: (1) Neurologist-confirmed MS and a maximum of two years have elapsed since the diagnosis; (2) has been in the same place at the time of MS and has lived for more than three years to measure Rn concentration, (3) Having informed consent to participate in the study, (4) Not having any congenital, metabolic or chronic disease other than MS based on patient reports, (5) Not having a family history of MS, and (6) Being in the age range of 50–20 years. Inclusion criteria for controls included: (1) Having informed consent to participate in the study, (2) Not having any congenital, metabolic, or chronic disease based on patient reports, (3) Having the same sex as the patient, (4) Not having a family history of MS, (5) Use of healthy people from the same neighborhood of the case group, and (6) Living in the same residential house with more than three years of experience. Due to the existing limitations and according to the pilot, at least 50 people were considered as the case group, of which 5 people were excluded from the study. The control group, which were matched in terms of sex, location, and the economic conditions of the study, were statistically selected twice as much as the case group. Finally, 45 patients with MS and 100 controls were evaluated. Thus, the total number of case and control samples was 145.

### Rn measurement

Rn gas data were collected using a CR-39 detector. The CR-39 dm used is the inactive solid-state nuclear track detector (SSNTD). SSNTD detectors are one of the best methods for measuring Rn gas for a long time due to their durability and strength, availability, and ease of use. Detectors used when exposed to ambient air record alpha particles produced by Rn gas as traces (grooves)^41^. The detectors were exposed to Rn gas in homes for 6 months, from September to February. The detectors were then sent to a laboratory to measure the concentration of Rn gas. A total of 145 houses were surveyed for Rn gas concentrations. Two detectors were placed in each house. Hence, 290 detectors were used in the study. One of the detectors was installed in the bedroom and the other in the living room.

### Quality assurance

Quality assurance of Rn gas measurement by the detector was performed using the detector calibration by the manufacturer during installation. Thus, after purchasing the detectors, they were calibrated by the company and installed in the desired location.

### Data collection related to food intakes

In this study, a semi-quantitative feed frequency (FFQ) questionnaire was used to assess food intake. The modified sample of the questionnaire was 178 items whose validity was evaluated in the Tehran Lipid and Glucose Study^39,40^. In this questionnaire, there were common foods used in Yazd along with a standard serving size for each food item. The subjects were asked to mention their consumption frequency of each food according to its amount in the previous year. Although the repetition of each food was intended for one year, depending on the type of food, the question was repeated depending on the repetition of consumption per day, week, or month. The mentioned values of each food were converted to gr/day using the guide of home scales^42^.

### Collection of other data

Authoritative articles in this field were used to prepare a general information questionnaire (economic and social status)^37^. Socio-economic status of individuals based on the data obtained from the socio-economic status assessment questionnaire on variables such as age (year), sex, marriage, family economic status (status of ownership, number of cars in the house and its type and total family income), smoking, medical and drug history and MS family histor, education, physical activity, stress level, and sun exposure rate were collected. Also, the height and weight of the participants were assessed qualitatively based on the latest measurements made by the individual.

### Ethic approval

This study was approved by the public health ethics committee of Shahid Sadoughi University of Medical Sciences and health services, Yazd; the ethic number: IR.SSU.SPH.REC.1396.24. Also, we confirm that all experiments were performed in accordance with relevant guidelines and regulations.

### Data analysis

After collecting information, data were analyzed with SPSS software version 2020. In the case of quantitative variables, before selecting the type of statistical tests, the normality was first examined using the Kolmogorov–Smirnov test. The α for Rn gas concentration was < 0.07 and other variables were > 0.07. Then, logistic regression and covariance (ANCOVA) tests were used to compare quantitative variables with normal distribution between case and control groups, and Non-Parametric, Mann–Whitney, and Kruskal–Wallis tests were used to compare abnormal quantitative variables. Chi-square test was also used to compare qualitative variables between case and control groups. If the chi-square hypotheses were not valid and > 20% of the expected homes were below 5, the Fisher test was used.

### Rn gas concentration analysis

To determine the relationship between building materials and Rn gas concentration and to determine the relationship between Rn gas concentration and MS, NonParametric and Kruskal–Wallis tests were used for more than three variables and the Mann–Whitney test was used for binary variables. Wilcoxon test was also used to analyze the dependent variables. The Chi-square test was used to determine the relationship between building materials and MS.

### Dietary pattern analysis

Foods items were grouped into 36 predefined food groups. In general, food grouping was done based on the similarity of food profiles. To identify the relationship between food groups and MS in the analyzes. Also, 95% confidence interval and Odd Ratio (OR) for the variables calculated in logistic regression were calculated. Comparison of means and ratios between the received tertiles from the food patterns extracted was performed by variance ANOVA and Chi-square, respectively. Logistic regression was used to find the relationship between received food groups and MS. Subjects were divided into tertiles according to food groups and nutrients received. The odds of developing MS in the second and third tertiles were calculated compared to the first tertile. Calculating the value of P for trend (P for trend) by considering the middle of each tertile as a continuous variable. Models used include Model I: Modified for age, gender, and energy, Model II: Modified for marriage, occupation, education, sun exposure, smoking, use of sunscreen, body mass index (BMI), spouse's education and occupation, and the economic situation, and model III: without adjusting the effect of the above distorters. Also in the adjusted model, the effect of age, sex, energy, marriage, economic status, education, occupation, smoking, sun exposure, sunscreen, BMI, education, and occupation of the spouse was considered. On the other hand, the comparison of the mean intake of dietary patterns as a model III and after adjusting the confounding and possible contexts variables was done using analysis of variance and covariance (ANCOVA) tests, respectively. In all analysis *P*-value < 0.05 was used for significance.

### Findings

In this study, external factors that may affect the incidence or severity of MS, such as exposure to Rn gas and intake of different food groups on the prevalence of MS have been investigated. To examine the information of the people included in the study, demographic information was examined. The demographic information of the study participants is given in Table 1.

**Table 1:** Demographic information of case and control groups.

	Case N = 45	Control N = 100	Total N = 145	*P *value
Age (year)	34.51 ± 8.53	40.14 ± 12.45	38.39 ± 11.64	0.002
Weight (Kg)	65.36 ± 12.04	68.03 ± 10.52	67.20 ± 11.04	0.225
BMI (Kg/m^2^)^a^	24.29 ± 4.19	25.32 ± 3.74	25.002 ± 3.90	0.197
Sex [n (20)]				0.216
Male	8 (17.8)	29 (29.0)	37 (25.5)	
Female	37 (82.2)	71 (71.0)	108 (74.5)	
Marital status [n (20)]				1.00
Single	8 (17.8)	17 (17.0)	25 (17.2)	
Married or widowed	37 (82.2)	83 (83.0)	120 (82.8)	
Education [n (20)]				0.858
Degree less than high school	20 (44.4)	46 (46.5)	66 (45.80)	
University or college	25 (55.6)	53 (53.5)	78 (54.2)	
Job [n (20)]				0.920
Government emPloyed	17 (40.5)	42 (42.0)	59 (41.5)	
Self employed	7 (16.7)	14 (14.0)	21 (14.8)	
HousekeePer or not emPloyed	18 (42.9)	44 (44.0)	62 (43.7)	
Economic statues [n (20)]^b^				0.031
Low income	12 (31.6)	27 (31.8)	39 (31.7)	
Middle income	17 (44.7)	20 (23.5)	37 (30.1)	
High income	9 (23.7)	38 (44.7)	47 (38.2)	
Smoking status [n (20)]				0.550
Current or ex-smoker	3 (6.7)	11 (11.0)	14 (9.7)	
Non-smoker	42 (93.3)	89 (89.0)	131 (90.3)	
Regular Physical [n (20)]				0.030
Yes	5 (15.6)	27 (84.4)	32 (100)	
No	40 (37.0)	68 (63.0)	108 (100)	
T otal	45 (32.1)	95 (67.9)	140 (100)	
Sun exPosure [n (20)]				0.009
Low 15 min	15 (34.1)	13 (13.3)	28 (19.7)	
5–15 min	19 (43.2)	38 (38.8)	57 (40.1)	
15–30 min	4 (9.1)	19 (19.4)	23 (16.2)	
UP 30 min	6 (13.6)	28 (28.6)	34 (23.9)	
Sun screen use [n (20)]				0.357
Yes	19 (43.2)	35 (35.0)	54 (37.5)	
No	25 (56.8)	65 (65.0)	90 (62.5)	
SPouse education [n (20)]				1.00
Degree less than high school	19 (51.4)	45 (51.7)	64 (51.6)	
University or college	18 (48.6)	42 (48.3)	60 (48.4)	
SPouse job [n (20)]				0.230
Government emPloyed	11 (30.6)	38 (44.7)	49 (40.5)	
Self employed	19 (52.8)	31 (36.5)	50 (41.3)	
HousekeePer or not emPloyed	6 (16.7)	16 (18.8)	22 (18.2)	

According to Table 1, the mean age of the subjects in the case and control group was 34.51 ± 8.53 and 40.14 ± 12.45. The mean BMI was 24.29 ± 4.19 for the case group and 25.002 ± 3.90 for the control. In terms of gender, there was no significant difference between the case and control groups due to matching (*P* = 0.216).

People with MS were significantly different from controls in terms of age, economic status physical activity, and sun exposure rate (*p* < 0.005). The subjects in the case group had lower age and physical activity and were less exposed to sunlight. Also, middle-income people in the case group were much higher than controls, on the other hand, controls had a higher income (*P* < 0.03). There was no statistically significant difference in terms of weight, BMI, marital status, education, occupation, smoking, sunscreen, and occupation of the spouse between the case and control groups (*p* = 0.197). Because the characteristics of the residence affect the amount of Rn gas, in this study, the effective properties of materials and other construction characteristics were investigated, which are given in Table 2.

**Table 2:** Details of the residential house of the participants.

Building characteristics	Number (Percent) MS	Number(Percent) Without MS
**Foundation**		
Concrete	15 (33.3%)	43 (43.0%)
The brick	26 (57.8%)	52 (52.0%)
Others	4 (8.9%)	5 (5.0%)
**Structure**		
Concrete	12 (26.7%)	38 (38.0%)
Metal	10 (22.2%)	36 (36.0%)
Reinforced concrete	6 (13.3%)	11 (11.0%)
Others	17 (37.8%)	15 (15.0%)
**Materials used in the facade**		
Rock	14 (32.6%)	14 (14.3%)
Brick clay	19 (44.2%)	60 (61.2%)
Ceramic	1 (2.3%)	12 (12.2%)
Others	9 (20.9%)	12 (12.2%)
**Floor covering**		
Ceramic and Tile	32 (71.1%)	50 (50.0%)
Mosaic	13 (28.9%)	41 (41.0%)
Others	0 (0.0%)	9 (9.0%)
**Wall covering**		
Ceramic and tile	11 (25.0%)	14 (14.0%)
Plaster and Rock	8 (18.2%)	12 (12.0%)
Plaster and Ceramic	11 (25.0%)	25 (25.0%)
Plaster and Color	13 (29.5%)	45 (45.0%)
Others	1 (2.3%)	4 (4.0%)
**Most used space**		
Living room	39 (86.7%)	86 (86.0%)
Room	3 (6.7%)	8 (8.0%)
Others	3 (6.7%)	6 (6.0%)
**Heating**		
Heater	30 (68.2%)	74 (74.7%)
Radiant	14 (31.8%)	22 (22.2%)
Others	0 (0.0%)	3 (3.0%)
**Cooling**		
Air conditioner	41 (91.1%)	82 (82.0%)
Air conditioner and Others	4 (8.9%)	18 (18.0%)
**Ventilation**		
No Ventilation	7 (15.6%)	0 (0.0%)
Natural ventilation	28 (62.2%)	75 (75.0%)
Fan and hood	10 (22.2%)	25 (25.0%)
**Window type**		
Normal	38 (86.9%)	88 (88.0%)
Two shells	7 (15.6%)	12 (12.0%)
**Kitchen type**		
Open	33 (73.3%)	73 (73.0%)
No open	12 (26.7%)	27 (27.0%)
**Kitchen ventilation**		
No Ventilation	5 (11.1%)	5 (5.0%)
Natural ventilation	9 (20.0%)	26 (26.0%)
Fan and hood	31 (68.9%)	67 (67.0%)
Others	0 (0.0%)	2 (2.0%)
**Number of floors**		
Underground	11 (24.4%)	13 (13.0%)
First floor	26 (57.8%)	78 (78.0%)
More than two floors	8 (17.8%)	9 (9.0%)
**Building age**		
Under 20 years old	30 (66.7%)	69 (69.0%)
20 years uP	15 (33.3%)	31 (31.0%)
**Building type**		
The aPartment	12 (27.3%)	15 (15.0%)
Villa	32 (72.7%)	85 (85.0%)

**Figure 1:**
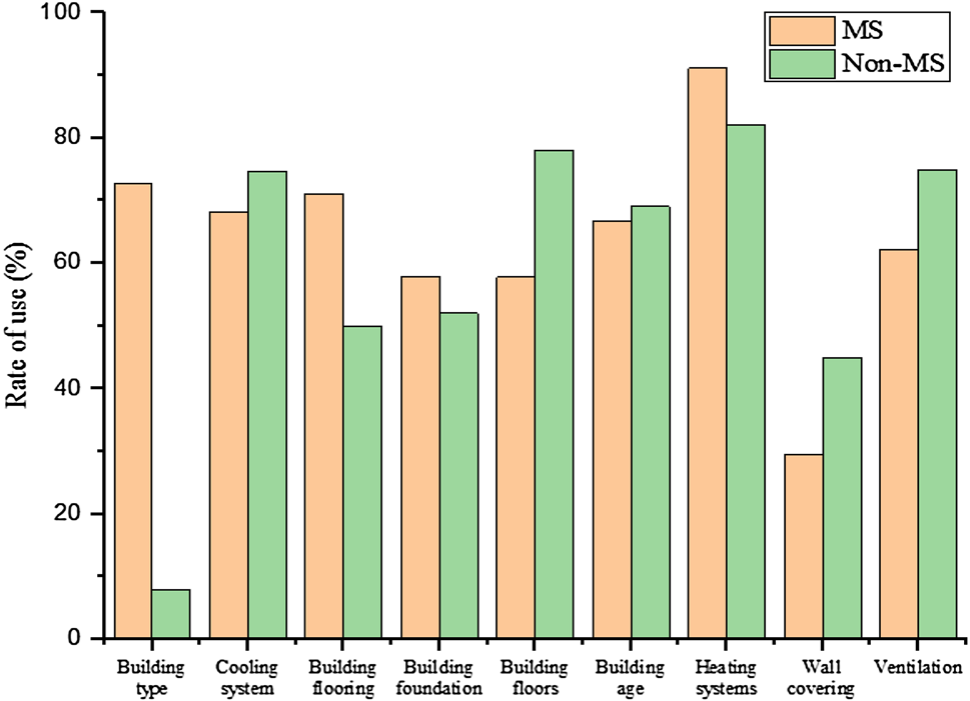
Construction characteristics and percentage of each type in the homes of MS patients and healthy people.

More brick was used in the foundation of residential houses, with a higher percentage belonging to the homes of people with MS (57.8%). Most of the materials used in the construction of buildings were concrete, which belonged to the homes of people without MS (38%). While in the homes of people with MS, other materials other than concrete, metal, and reinforced concrete were used for the structure (37.8%). Also, the use of pottery bricks in the facade of the building accounted for the highest percentage, with a higher percentage related to the homes of people without MS (61.2%) (Table 2). The construction characteristics and the percentage of each type in the homes of MS and healthy individuals are given in Fig. 1.

**Figure 2:**
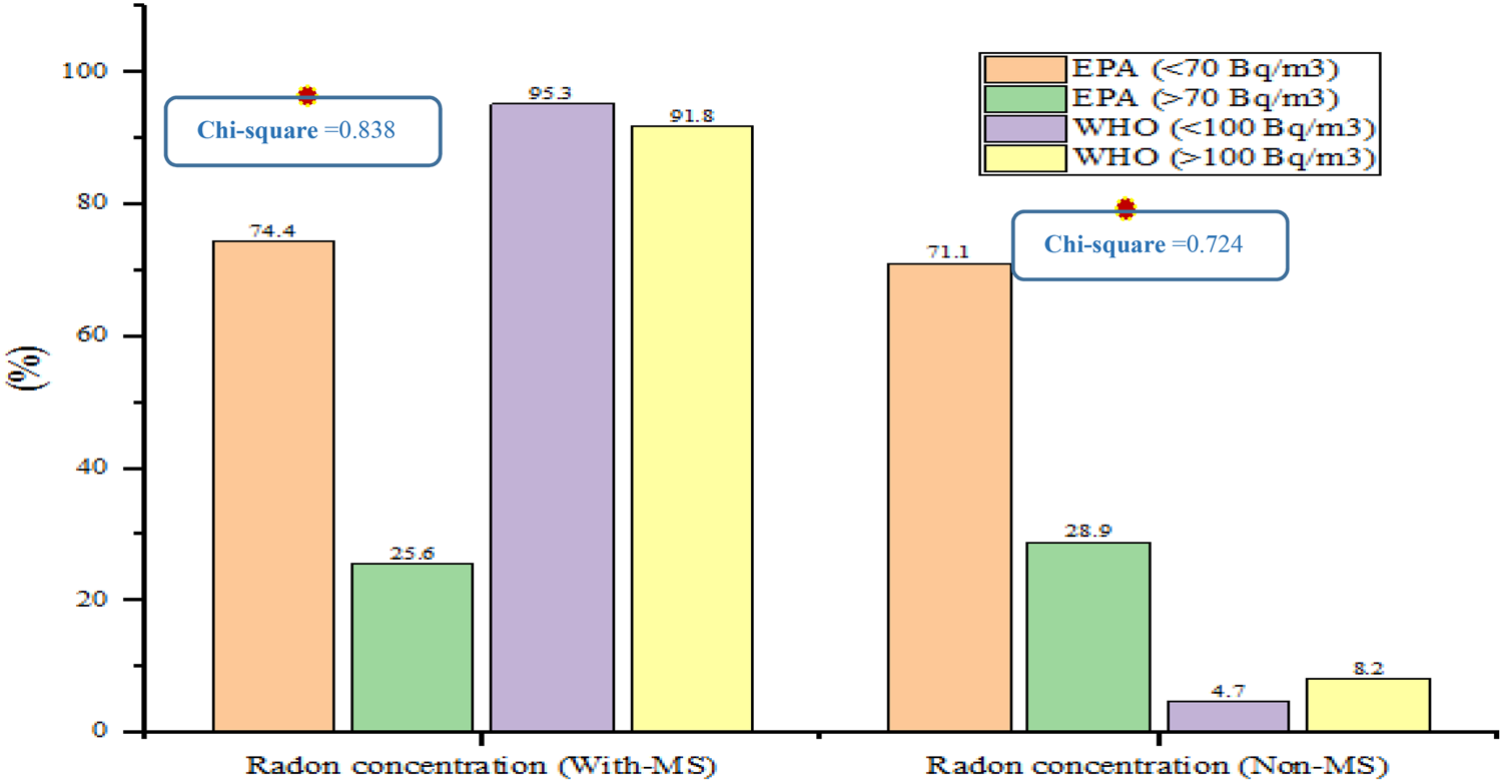
Total Rn gas concentration in comparison with EPA and WHO standards.

The materials used in the flooring of residential houses were more than ceramic tiles, which was a higher percentage related to the homes of people with MS (71.1%). A higher percentage of house walls of paint and plaster was related to non-MS (45%) and more use of heaters for home heating was related to non-MS homes (74.7%). In residential houses, air conditioners are mostly used to cool the house and in the houses of people with MS (91.1%). Also, more natural ventilation was used for air circulation in the homes of non-MS people (75%) and windows in residential houses were more than the normal type (88%). A higher proportion of non-MS people lived on the first floor or ground floor (78%). The age of homes for non-MS people was < 20 years (69%). Regarding the age of 20 years in the homes, the patients accounted for 33.3% of the cases, and in the homes of the non-infected people 31% of the cases. Most non-MS people lived in villas (85%) (Fig. 2). Rn concentrations in the homes of participants in comparison with EPA and WHO gidelines are shown in Fig. 2.

According to Fig. 2, no significant relationship was found between Rn gas concentration in comparison with EPA and WHO standard with MS (Chi-square > 0.005). Nevertheless, Fig. 2 shows what percent of homes with MS and non-MS had Rn concentrations higher and lower than the EPA standard (70 Bq/m^3^) and what percent had higher and lower than the WHO standard (100 Bq/m^3^). For example, 91.8% of the homes of people with MS and 8.2% of non-MS had an Rn higher than the WHO standard (100 Bq/m^3^). Moreover, mean dietary intake between MS and non-MS participants showed average consumption of meat products, offal meats, poultry, eggs, butter, fruits and juices, vegetables, refined grains, mayonnaise, nuts, sweets, hydrogenated fats, sugar, carbonated beverages, and red meat was higher in the case group. The average consumption of fish, low-fat and high-fat dairy products, tomatoes, potatoes, whole grains, buttermilk, pickles, and legumes was higher in non-MS patients (Table S1 and S2). On the other hand, the mean of nutrient intake between participants with MS and non-MS showed that the mean of total fat, saturated fats, protein, and carbohydrates was higher in MS people (*P* < 0.05) (Table S3).

### Relationship between Rn gas concentration and building ventilations

Total Rn had a significant relationship with cooling devices (*P* = 0.021). Thus, if a cooler was used to cool the house, there was a lower total Rn concentration than other cooling devices (fans, packages, etc.). Homes that used the fan and hood simultaneously for kitchen ventilation had lower Rn concentrations, which was statistically significant (*P* = 0.053). Buildings older than 20 years had higher Rn concentrations than buildings younger than 20 years (*P* = 0.038). However, in buildings that used ceramic bricks for building facades, mosaics for flooring, and plaster and ceramics as wall coverings and heating devices such as heaters and double-glazed windows, the total Rn concentration was higher than in other materials. Also, buildings that were not ventilated and lived on the lower floors and basements had higher Rn concentrations (Table S4).

### Relationship between MS and building materials

The materials used in building construction have a significant relationship with MS (*P* = 0.015). Also, the materials used in the facade of the building had a significant relationship with it (*P* = 0.010). The materials used in the flooring of the building had a significant relationship with MS (*P* = 0.021). Upstairs compared to the basement was significantly associated with reduced risk of MS (*P* = 0.044) (Table S5).

### Correlation of Rn gas concentration with MS

**Figure 3:**
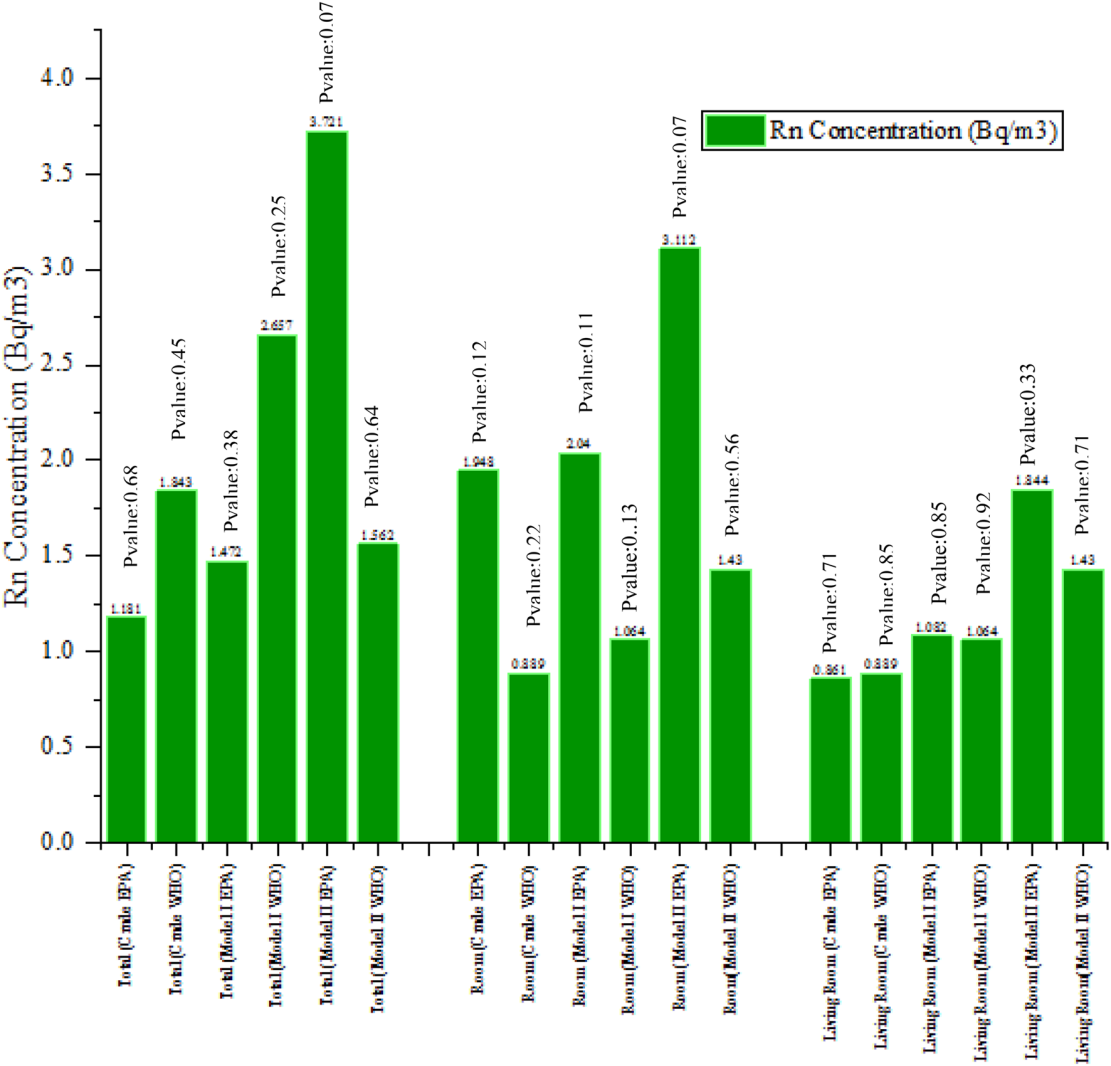
Correlation of Rn gas concentration with MS.

The relationship between Rn concentration and MS is shown in Fig. 3.

Based on the results of statistical analysis, shown *P*-value in Fig. 3, the concentration of total Rn gas was higher in the homes of studied people. On the other hand, Rn gas concentration in the room, living room, and the whole house according to EPA and WHO criteria were not significantly associated with the chance of MS (*P* > 0.005) (Fig. 3).

### Relationship between food group intake and MS

People in the case group used more processed meat than the control (*P* < 0.001). After adjusting the effect of possible confounding variables in models 1 and 2, this relationship remained significant (*P* < 0.004). Individuals in the case group consumed more butter than those in the control group, which was significant in Model III (*P* < 0.04). However, after adjusting the variables, it was not significant (*P* > 0.05). Tomato consumption after modulating confounding variables in the model I was significantly higher in the case group (*P* < 0.03). But in model II, it was out of the significant state. In the case group, fewer potatoes were consumed than the control group (*P* < 0.04) after adjusting for confounding variables remained significant (*P* < 0.02). In the case group, fewer potatoes were consumed than the control group (*P* < 0.04) after adjusting for confounding variables, this relationship remained significant (*P* < 0.02). Also, the consumption of sweets in the case group was higher than the control group and showed a significant relationship (*P* < 0.007) that after adjusting the confounding variables in models I and II, this relationship remained significant (*P* < 0.01). The controls consumed fewer drinks than the subjects in the case group, which showed a significant relationship (*P* < 0.001), which remained significant after adjusting for confounding variables in models I and II (*P* < 0.04) (Table S6).

### Relationship between mean food intake and MS

Red meat consumption was higher in the case group than in the control, which was a significant relationship in Model III (*P* < 0.02), but after adjusting the variables, it was not significant (*P* > 0.05). Consumption of saturated fatty acids in Model III was lower in the control group than in the case group and showed a significant relationship (*P* < 0.05), but after adjusting for confounding variables, it was significant (*P* > 0.05). On the other hand, the consumption of unsaturated fatty acids with a double bond in the case group was higher than the control (*P* < 0.001). After adjusting for confounding variables in models I and II, this relationship remained significant (*P* < 0.01) and there was no significant relationship between the means of intake in other food groups.

The chance of getting MS in the highest tertile compared to the lowest tertile received in groups showed that the chance of getting MS in people who are in the highest tertile in terms of receiving processed meat was 5 times higher than those who receive less processed meat. This relationship remained significant after adjusting for confounding variables (*P* < 0.001). Also, people who received the highest tertile in terms of potato and pickle intake were less likely to develop MS than those who received more of this food group. Also, an inverse relationship was found between refined grains intake and MS after adjusting for confounding variables in Model I, although this relationship was significant in the third tertile consumption compared to the first tertile (OR 0.27, 95% CI 0.09, 0.78). Hence, people who were in the second (OR 2.88, 95% CI 1.08, 7.68) and the third (OR 4.08, 95% CI 1.60, 10.40) from receiving carbonated and sweetened beverages, compared to people who received the lowest intake, had a higher developing MS chance.

The developing MS chances comparison in the highest tertile to the lowest showed that people who were in the third tertile (OR 2.71, 95% CI 1.10, 6.69) received the most saturated fatty acids than those who received the lowest. People who received the highest tertile dual-bond unsaturated fatty acids were more likely to develop MS than those who received the lowest tertile. People in the third tertile (OR 2.49, 95% CI 1.01, 6.16) had a higher chance of developing MS than those with the lowest dual polyunsaturated fatty acids. This relationship was out of significance after adjusting the confounding variables in models I and II. People who were in the highest tertile in terms of fiber intake after adjusting for confounding variables in Model 1 were less likely to develop MS than those in the lowest tertile.

### Interaction of each food group with Rn gas concentration in residential houses

The food group interaction with Rn gas concentration are shown in Table 3.

**Table 3:** Interaction of each food group with Rn gas concentration in residential houses.

Food groups	Low Rn concentrations OR(95%CI)	High Rn concentration OR(95%CI)
**Fruit**		
T1	6.36 (0.78, 51.59)	3.96 (0.57, 27.78)
T2	5.85 (0.85, 40.36)	3.87 (0.48, 31.32)
T3	1	0.41 (0.04, 3.97)
**Potato**		
T1	3.48 (0.45, 26.84)	1.16 (0.18, 7.64)
T2	2.86 (0.41, 20.13)	1.70 (0.18, 4.31)
T3	1	0.47 (0.05, 4.31)
**Pickles**		
T1	117.11 (6.57, 2088.4)	24.31 (1.74, 339.84)
T2	23.60 (1.86, 298.69)	23.95 (1.42, 404.20)
T3	1	3.53 (0.25, 50.60)
**Refrains grain**		
T1	7.03 (0.91, 54.11)	9.20 (1.27, 66.64)
T2	9.27 (1.11, 77.42)	2.63 (0.33, 21.01)
T3	1	2.62 (0.25, 27.22)
**Fiber**		
T1	12.40 (1.09, 141.58)	3.97 (0.52, 30.45)
T2	6.69 (0.63, 65.63)	4.07 (0.46, 35.59)
T3	1	0.69 (0.08, 6.19)
**Process meat**		
T1	1	0
T2	0.43 (0.8, 2.37)	0
T3	5.73 (1.09, 29.95)	4.8 (1.10, 20.91)
**Total fat**		
T1	1	1.13 (0.20, 6.50)
T2	0.26 (0.03, 2.09)	1.33 (0.20, 8.87)
T3	4.41 (0.43, 44.97)	9.26 (0.81, 106.00)
**Soft drink**		
T1	1	1.19 (0.21, 6.80)
T2	0	1.43 (0.29, 6.91)
T3	1.40 (0.21, 9.12)	2.44 (0.040, 15.03)
**Saturated fat**		
T1	1	0.52 (0.09, 3.06)
T2	0.40 (0.05, 3.09)	0.89 (0.16, 4.85)
T3	1.74 (0.26, 11.65)	2.34 (0.32, 17.08)
**Monounsaturated fat**		
T1	1	2.19 (0.33, 14.52)
T2	0.49 (0.06, 4.25)	0.93 (0.14, 6.38)
T3	8.36 (0.84, 82.97)	18.75 (1.73, 202.59)
**Polyunsaturated fat**		
T1	1	1.21 (0.22, 6.46)
T2	0.44 (0.06, 2.94)	0.35 (0.04, 3.02)
T3	0.94 (0.14, 6.31)	2.95 (0.45, 19.27)

According to the current study, people who had moderate to low levels of pickles and were less exposed to Rn had a higher chance of developing MS than those who had the least exposure to Rn and the most pickles. Therefore, it can be said that low and moderate consumption of pickles may increase the risk of MS. People who had lower exposure to Rn gas and a moderate intake of refined grains had a higher chance of developing MS than people who had the highest intake of refined grains and lower exposure to Rn gas. Also, the risk of developing MS in people who consumed refined grains at low levels but had higher exposure to Rn but was higher than in the reference group.

Therefore, low consumption of refined grains along with exposure to Rn gas increases the risk of MS. People with the lowest fiber intake and lower exposure to Rn were more likely to develop MS than those with the highest fiber intake and lower Rn exposure. As a result, consuming less fiber increases the risk of developing MS. Also, people who received a higher intake of meat products and lower exposure to Rn gas had a higher chance of getting heat than those who received the least intake of meat products and had lower exposure to Rn gas. Finally, it can be said that high consumption of meat products increases the risk of MS. In other words, high levels of double-bonded unsaturated fats and high exposure to Rn gas can increase the risk of MS. At different levels of Rn and fruit and potato intake, the chances of developing MS were not significantly different from those with the highest intake of fruit and potato and the least exposure to Rn gas. Also, in other cases, there was no significant difference in different levels of Rn gas and food intake compared to the reference group.

## Discussion

The Rn gas concentration in homes is affected by various factors such as ventilation, type of building, etc., and this gas as an indoor air pollutant can have adverse effects on health. In the present study, the effect of building characteristics on Rn gas concentration, food intake in people with MS and non-MS, and their interaction was investigated. The results showed that there is a significant relationship between economic status (*P* = 0.031), physical activity (*P* = 0.030), and sun exposure (*P* = 0.009) with MS risk reduction. Some studies have reported a statistically significant difference between the income of the group that used complementary therapies and the group that did not use these therapies (*P* = 0.05)^43^. In contrast, the results of a factor-analytical study in the United States showed a positive relationship between annual family income and MS incidence (*P* < 0.0001; r = + 0.57)^44^. In the WHO Atlas, charts and figures show that is more prevalent in higher-income countries and continents^16^.

However, in less developed countries, access to diagnostic facilities is less than in developed countries^45^. In Tasmania, the highest exposure to sunlight (average 2–3 h or more during the day, weekends, and holidays) over 6–15 years has been associated with a reduced risk of MS^46^. High levels of vitamin D and exposure to sunlight are associated with a reduced risk of MS^47^. Increased exposure to sunlight and vitamin D had an effect on reducing the progression of MS (superiority ratio = 0.24; 95% confidence interval = 0.38–0.15; *P* = 0.002)^48,49^. At least two biological mechanisms have been proposed that suggest the sun's UVR may reduce the MS risk. The first mechanism, the sun's UVR, may have a protective effect on the immune system, which is a proven effect on the immune system in rodents^50^. Hence, increased sunlight in humans inhibits T cell activity^51^. The second mechanism may be through the involvement of solar UVR in the biosynthesis of vitamin D, which produces vitamin D through the chemical reaction of photolysis in human skin, and UVR acts as a catalyst^52^.

In the present study, physical activity reduced the incidence of MS. The results of a study showed that people with MS had less physical activity than non-MS^53^. Also, in a study that examined physical activity in people with MS, the spent time on moderate to severe physical activity at 13 min was significantly different between case and control^54^. This can increase the risk of cardiovascular disease, which affects the rate of disability^53^. Physical activity reduces plasma leptin levels and decreases leptin receptor genes in the liver. In addition to acting on the central nervous system, leptin affects peripheral tissues such as the liver to protect against fat accumulation. The results of studies in humans and animals indicate that physical exercise increases the reduction of blood lipids by lowering plasma leptin levels^55^.

Rn gas concentration depends on factors such as building materials, temperature, humidity, air turbulence, airflow, ventilation rate, geology, lifestyle, meteorological conditions, and physical ways of infiltration^56–58^. In the case of indoor air, studies have shown that the risk of exposure is higher for poorly ventilated buildings^59,60^. The study by Kumar et al. showed that Rn concentrations strongly depend on ventilation^61^. In Netherlands, where Rn concentrations were estimated using calculations of two models, ventilation caused changes in Rn levels^62^. According to the results of current study, residential houses that were not ventilated and used heating devices with heaters and double-glazed windows, although not significant, had a higher concentration of Rn gas in them.

A study in Hong Kong showed that the cooling system and the age of the building affect the amount of Rn^63^. A cross-sectional study showed a significant relationship between building age and Rn concentration (*P* < 0.001), which was higher in older homes^64^. Brick houses have more Rn than concrete, brick, and plaster^65^. A study in winter and summer showed that there is no significant relationship between Rn and building age, but Rn concentration in houses over 55 years decreases with building age^66^.

The use of brick walls in comparison with concrete walls increases the amount of Rn depending on the building by 20 and stone walls by 72%. In contrast, wooden walls reduce the share of building-dependent Rn by 57% compared to brick walls^67^. The average concentration of Rn gas in buildings with clay and mud (40 ± 70) and brick (16 ± 29) is higher than in concrete buildings^68^. This is because concrete is compact and does not allow Rn to easily exit and enter the environment, while clay and brick have low compaction, high porosity, and high dispersion of Rn gas^69^. Building materials used in home decoration tiles, ash, and granite slabs have higher Rn emissions than other^70^. Materials such as gypsum and tiles have very little Rn^71,72^. So, the radiation of natural Rn beams in closed spaces can be due to materials used in building materials^73^. Because the range of alpha particles in solids is a few microns, proper staining or the use of wallpaper can greatly reduce the exposure of residents^68^. In general, due to the difference in the concentration of radionuclides in building materials, the geological conditions of the region^74,75^, the difference in the nature of samples and radium content of samples due to differences in radium around the world^76^ and construction industry usage from wastes of other industries^77^ the amount of Rn released from materials can vary.

Soil Rn from the top to down is influenced by factors such as gas emissions, airflow, and radioactive half-life^58^. Because the basement is surrounded by soils, which is the main source of Rn gas entering the ambient air, basements have higher concentrations of Rn gas than upper floors^68^. A study showed that the concentration of Rn in each floor from the basement to the upper floors decreased by 15 to 20%^78^. Rn concentrations in three-story apartments in Belgrade-Serbia were approximately 20% lower per floor^79^. The concentration of Rn in the basement is 2 times that of the earth^80^ and > 30% of the basements have a Rn concentration > 148 Bq/m^3^, which is consistent with the results obtained in the present study^81^.

No significant relationship was found between total Rn gas concentration and MS in Yazd, but the total Rn gas concentration was higher. A retrospective study of the incidence of MS and its association with Rn concentrations showed that although an increase in the prevalence of MS with increasing Rn concentrations has been identified, there is no significant correlation. The association between MS and Rn concentrations, although not statistically significant, was found to increase the likelihood of MS prevalence by increasing each unit over the average remaining Rn exposure time^82^. In contrast, there was a significant positive correlation (*P* < 0.01) between indoor Rn concentration and magnesium levels in the air in MS patients. According to one hypothesis, the Rn content in inhaled air is an important factor in MS prevalence^11^.

According to a study, patients in Iran use solid vegetable oils before the development of MS^83^. The association between an increased risk of MS and high consumption of animal fats was first reported by Swank^84^. The results of epidemiological studies have been contradictory association between food and MS^85–87^. An investigation showed that the risk of MS increases with saturated fats (*P* < 0.001) and meat (*P* = 0.002) consumption^88^. Iincreasing the energy intake (*P* = 0.004), carbohydrates (*P* = 0.005), fats (*P* = 0.043) and multi-band fatty acids (*P* = 0.001) is significantly associated with an increased risk of MS^89^. People who consumed the highest tertile of solid oil and carbonated beverages were 1.58 and 1.87 times more likely to develop MS, respectively (*P* < 0.05)^90^. Also, a diet high in animal fats or saturated fats, and a low intake of double-bonded fatty acids or omega-3 fatty acids may increase the risk of MS^91^. In the present study, the increase in unsaturated fats with a double band consumption was directly related to the chance of developing MS, which contradicted the results of studies in this field. The lack of change could be because of study type, which was retrospective, patients changed their diet after diagnosis, which can affect the results of the study.

Data from experimental studies have shown that excessive saturated fatty acids intake as an effective factor in the demyelination of neurons can lead to the progression of the disease^92–94^. The consumption of saturated fatty acids may lead to blockage of capillaries in the central nervous system and reduced flexibility of the vessel wall through the accumulation of red blood cells and platelets, which can eventually lead to demyelination of nerves by developing hypoxia^88,95^. Consumption of meat and its products (*P* = 0.001)^14^ and animal fats, mutton and beef (*P* = 0.017) and dried meat, and sausages (*P* = 0.007) are associated with an increased risk of MS^94,96^. In contrast, in two large cohort studies in women, researchers noted a lack of association between dairy, poultry, fish, red meat, and processed meat intake and the risk of MS^85,97^. Nitrate in various forms as preservatives in meat products may be a biological argument in the pathogenesis of autoimmune processes. That is, nitrous oxide with meat proteins may cause severe oxidative damage to biological tissues^98^.

In a study^99^ based on the Mediterranean diet, it was shown that whole and refined grains were inversely related to the risk of MS. Contrary to these findings, a case–control study showed a positive relationship between refined grains and MS risk^87^. Before being diagnosed with MS, MS patients ate large amounts of white bread, more sugars, and carbonated beverages^86^ and less bread, cereals, and some vegetables and fruits^100^. Whole grains are a good source of fiber. The role of fiber in preventing chronic diseases such as MS is based on their bioactive substances with antioxidant and anti-cancer properties, especially bran^101^. Also, the high consumption of carbonated beverages due to their high phosphate content can affect calcium absorption. Calcium has important effects on the synthesis of myelin sheath lipids and the immune system, changes in calcium concentration can affect its function in the immune system. Findings have highlighted the relationship between low calcium intake and the chance of developing MS^14^.

Vegetables consumption reduces the risk of MS and consumption of various fruits and vitamin C^102^ have a protective effect against MS^14^. A study in Croatia reported that the prevalence of MS was inversely related to the daily consumption of fresh fruits and vegetables^94^. The protective effect of plant protein and fiber on the incidence of MS was reported (*P* = 0.004)^88^. Contrary to these findings, Zhang et al. Reported that no significant association was found between fruits and vegetables and an increased risk of MS^85^. Fruits contain many essential compounds such as phenols, vitamins, and minerals that can have oxidative effects. Vitamins, especially antioxidants, can neutralize free radicals and prevent lipid peroxidation in the white matter of the brain and demyelination of central nerves in MS^14,103,104^. Also, the antioxidant properties of polyphenols and carotenoids affect the restoration of oxidative balance. Certain polyphenols such as catechins and quercetin have anti-inflammatory and immunizing properties^27^.

### Study limitations

In all studies and research, there are always limitations that will not be ineffective in the process and results of research. The following are specific cases of limitations in the present study:

### Restrictions beyond the authority of the researcher

Some participants are likely to be influenced by their personality in completing the questionnaire and giving the correct information, although they are assured that the results will remain strictly confidential. Poor knowledge and low information of the research community on the subject of research, which of course also affects the response to questionnaires. Since the present study was retrospective, information about the dietary intake of individuals during the past year (for the case group the year before the disease, and the control group the year before the interview) was assessed using the FFQ questionnaire, which here is a possibility of Recall Bias in it. According to patients' memory, however, accurate reporting of normal eating habits was very difficult. On the other hand, the physiological condition of patients when filling out the questionnaire can affect their response.

Because food intake was completed by the individual through the FFQ questionnaire, there is a possibility of error in answering the questions. Where participants did not respond with confidence or were unable to recall specific information and may have reported a healthier diet than their eating habits. The required information regarding confounding variables such as physical activity reported by individuals was assessed using only two simple questions included in the general information questionnaire. Hence, this method can not be a very reliable indicator for assessing the status of this variable compared to the data obtained from a specific and valid questionnaire to assess the level of physical activity.

### Limitations available to the researcher

Lack of access to sufficient content on research-related variables and not having enough money to buy detectors were among the most important limitations available to the researcher.

Finally, due to the short period of the present study and the mentioned limitations, the findings of the present study cannot be generalized to communities, and there is a need for further studies to confirm or reject the results of this study.

## Conclusion

The results of study showed that the concentration of Rn gas in people with and without MS has no significant relationship and supports the possible role of some food groups in the prevention of MS. Only there was an interaction in the case of double-bonded unsaturated fats and refined grains with MS prevalence. Due to the fact that the etiology of MS is still unknown and a set of factors play an important role in disease occurrence, it is better to conduct such studies in other provinces with different climatic conditions and food behaviors in similar populations with larger sample sizes. Natural radioactive knowledge in building materials is more important than assessing possible radiological hazards to human health, developing standards and guidelines for the use and management of these materials. Also, very few measurements have been made in Iran about the amount of inhaled Rn. Therefore, it is recommended to do more research on the amount of Rn inhalation in various ways, especially building materials used in Iran.

## Supplementary Information

Supplementary Information.
