# Supplementary material for: Interaction of MS prevalence, radon gas concentration, and patient nutrition: a case–control study
Source: Sci Rep. 2021 Sep 9;11:17906. doi: 10.1038/s41598-021-96816-4 (PMC8429741; doi:10.1038/s41598-021-96816-4)
Supplement: Supplementary file 1 — Supplementary Information. [file 41598_2021_96816_MOESM1_ESM.docx]

Table S1: Radon gas concentration according to EPA and WHO standards

|  | **Number(Percent)MS** | **Number(Percent)**  **Without MS** | | **Chi-square** |
| --- | --- | --- | --- | --- |
| Radon concentration of living room _ EPA |  |  | 0.839 | |
| Under 70 Bq/m^3^ | 31(70.5%) | 72(73.5%) |  |  |
| UPPer 70 Bq/m^3^ | 13(29.5%) | 26(26.5%) |  |  |
| Radon concentration of room _ EPA |  |  | 0.162 | |
| Under 70 Bq/m^3^ | 34(79.1%) | 64(66.0%) |  |  |
| UPPer 70 Bq/m^3^ | 9(20.9%) | 33(34.0%) |  |  |
| Total Radon concentration_ EPA |  |  | 0.838 | |
| Under 70 Bq/m^3^ | 32(74.4%) | 69(71.1%) |  |  |
| UPPer 70 Bq/m^3^ | 11(25.6%) | 28(28.9%) |  |  |
| Radon concentration of living room_ WHO |  |  | 1.00 | |
| Under100 Bq/m^3^ | 40(90.9%) | 90(91.8%) |  |  |
| UPPer100 Bq/m^3^ | 4(9.1%) | 8(8.2%) |  |  |
| Radon concentration of room_ WHO |  |  | 0.344 | |
| Under100 Bq/m^3^ | 41(95.3%) | 86(88.7%) |  |  |
| UPPer100 Bq/m^3^ | 2(4.7%) | 11(11.3%) |  |  |
| Total Radon concentration_ WHO |  |  | 0.724 | |
| Under100 Bq/m^3^ | 41(95.3%) | 89(91.8%) |  |  |
| UPPer100 Bq/m^3^ | 2(4.7%) | 8(8.2%) |  |  |

Table S2: Average intake of food groups of people with MS and non-MS

|  | Case | Control |
| --- | --- | --- |
| Processed meat (g/day) | 14.27±2.72 | 1.33±1.82 |
| Organ meats (g/day) | 9.85±2.68 | 5.66±1.80 |
| Fish (g/day) | 9.84±2.40 | 11.87±1.61 |
| Poultry (g/day) | 61.32±13.16 | 59.27±8.83 |
| Eggs (g/day) | 28.74±3.16 | 22.53±2.12 |
| Butter (g/day) | 3.85±0.64 | 2.30±0.43 |
| Margarine (g/day) | 1.30±0.40 | 0.61±0.27 |
| Low-fat dairy Product (g/day) | 78.75±20.35 | 83.37±13.65 |
| High-fat dairy Product (g/day) | 164.68±37.97 | 189.11±25.47 |
| Tea (g/day) | 357.73±54.81 | 404.49±36.77 |
| Coffee (g/day) | 6.95±1.89 | 4.91±1.27 |
| Fruit (g/day) | 880.79±140.38 | 846.35±94.17 |
| Fruit juice (g/day) | 128.51±24.76 | 79.37±16.61 |
| Tomatoes (g/day) | 63.93±21.53 | 105.16±14.44 |
| Vegetables (g/day) | 238.41±33.00 | 208.77±22.14 |
| Garlic (g/day) | 0.59±0.23 | 0.86±0.15 |
| Potatoes (g/day) | 15.57±4.72 | 27.26±3.16 |
| Whole grain (g/day) | 60.41±12.78 | 72.50±8.57 |
| Refrains grain (g/day) | 269.44±30.74 | 253.82±20.62 |
| Snakes (g/day) | 4.80±2.09 | 7.37±0.40 |
| Dried fruit (g/day) | 10.79±2.37 | 10.50±1.59 |
| Mayonnaise (g/day) | 3.71±0.89 | 1.99±0.60 |
| Nuts (g/day) | 19.10±2.61 | 16.77±1.75 |
| Olive (g/day) | 6.65±1.60 | 4.21±1.07 |
| Sweet and dessert (g/day) | 64.82±7.20 | 40.97±4.83 |
| Hydrogenated fats (g/day) | 9.98±1.50 | 2.51±1.00 |
| Vegetable oil (g/day) | 8.55±1.75 | 7.65±1.17 |
| Sugars (g/day) | 32.71±4.76 | 26.28±3.20 |
| Condiment (g/day) | 28.60±3.80 | 23.99±2.55 |
| Soft drink (g/day) | 83.30±11.69 | 28.80±7.84 |
| Yogurt drink (g/day) | 60.78±22.30 | 88.02±14.96 |
| Broth (g/day) | 41.73±14.10 | 45.21±9.46 |
| Salt (g/day) | 6.67±1.18 | 6.47±0.79 |
| Pickles (g/day) | 13.34±12.09 | 28.31±8.11 |
| Legumes (g/day) | 32.68±8.77 | 44.08±5.88 |
| Red meats (g/day) | 85.77±8.30 | 62.17±5.57 |

Table S3: Mean nutrient intake of people with MS and non-MS

|  | Case | Control |
| --- | --- | --- |
| Energy (Kcal) | 3038.73±197.17 | 2636.61±132.27 |
| Total fat (g/day) | 111.61±6.82c | 82.44±4.58 |
| Saturated fat (g/day) | 31.15±2.18 | 25.22±1.46 |
| Mono unsaturated fat (g/day) | 34.87±2.24 | 24.08±1.50 |
| Poly unsaturated fat(g/day) | 42.96±7.79 | 24.93±5.22 |
| Protein (g/day) | 116.61±9.41 | 103.79±6.31 |
| Carbohydrate (g/day) | 430.84±33.01 | 396.28±22.15 |
| Dietary fiber intake (g/day) | 28.38±0.99 | 28.05±2.01 |

Table S4: Relationship between radon concentration and building materials in residential houses of participants

| Total Radon concentration  Median(min, max) | **N** | **Radon concentration of room**  **Median(min, max)** | **N** | **Radon concentration of living room**  **Median(min, max)** | **N** | **Building characteristics** |
| --- | --- | --- | --- | --- | --- | --- |
|  |  |  |  |  |  | **Foundation** |
| 62.24(45.42,179.26) | 55 | 63.99(38.1,177.12) | 55 | 62.21(40.36,181.4) | 56 | **Concrete** |
| 67.24(39.66,146.04) | 76 | 69.44(45.42,159.23) | 76 | 68.86(6.83,132.84) | 77 | **The brick** |
| 68.99(47.08,95.71) | 9 | 66.01(48.56,88.7) | 9 | 69.9(45.59,102.71) | 9 | **Others** |
| 0.110 |  | 0.506 |  | 0.045 |  | **P value** |
|  |  |  |  |  |  | **structure** |
| 65.54(45.42,179.26) | 49 | 64.09(46.55,177.12) | 49 | 64.6(40.52,181.4) | 49 | **Concrete** |
| 65.46(39.66,115.6) | 44 | 71.9(38.1,120.73) | 44 | 67.46(6.83,110.46) | 45 | **Metal** |
| 63.83(54.33,143.21) | 15 | 68.3(51.76,159.09) | 15 | 66(40.36,127.33) | 16 | **reinforced concrete** |
| 64.85(47.08,123.35) | 32 | 65.37(46.23,130.60) | 32 | 67.03(39.69,116.09) | 32 | **Others** |
| 0.885 |  | 0.635 |  | 0.974 |  | **P value** |
|  |  |  |  |  |  | **Materials used in the facade** |
| 64.28(51.12,115.59) | 26 | 64.17(49.67,120.16) | 26 | 65.7(40.36,111.02) | 26 | **Rock** |
| 67.53(39.66,179.26) | 78 | 71.72(45.42,127.12) | 78 | 66.51(6.83,181.40) | 79 | **Brick clay** |
| 61.86(47.08,146.04) | 13 | 60.89(48.56,159.23) | 13 | 62.9(45.42,132.84) | 13 | **ceramic** |
| 64.42(49.66,143.21) | 19 | 61.76(38.1,159.09) | 19 | 68.26(43.72,127.33) | 20 | **Others** |
| 0.541 |  | 0.187 |  | 0.712 |  | **P value** |
|  |  |  |  |  |  | **Floor Covering** |
| 65.39(45.42,179.26) | 78 | 64.74(38.1,177.12) | 78 | 65.99(40.36,181.4) | 80 | **Ceramic and Tile** |
| 67.07(39.66,127.98) | 53 | 68.99(46.55,127.74) | 53 | 65.27(6.83,128.23) | 53 | **Mosaic** |
| 63.83(58.48,89.13) | 9 | 66.17(51.76,88.71) | 9 | 65.14(52.68,89.56) | 9 | **Others** |
| 0.921 |  | 0.795 |  | 0.925 |  | **P value** |
|  |  |  |  |  |  | **Wall covering** |
| 63.47(49.2,146.04) | 25 | 6304(46.23,159.23) | 25 | 65.92(45.42,132.84) | 25 | **Ceramic and tile** |
| 61.29(50.15,123.35) | 20 | 63.59(49.09,130.6) | 20 | 63.69(43.72,116.09) | 20 | **Plaster and Rock** |
| 68.05(39.66,113.73) | 34 | 68.15(46.55,127.36) | 34 | 64.77(6.83,102.71) | 35 | **Plaster and Ceramic** |
| 66.18(45.42,179.26) | 55 | 68.99(38.1,177.12) | 55 | 65.7(39.69,181.4) | 56 | **Plaster and Color** |
| 65.33(58.07,76.06) | 5 | 68.65(58.93,74.16) | 5 | 69(52.15,77.95) | 5 | **Others** |
| 0.748 |  | 0.733 |  | 0.818 |  | **P value** |
|  |  |  |  |  |  | **Most used sPace** |
| 65.09(39.66,179.26) | 121 | 65.77(38.1,177.12) | 121 | 64.09(6.83,181.4) | 122 | **living room** |
| 75.15(53.89,127.98) | 10 | 71.67(46.55,127.74) | 10 | 71.82(61.22,128.23) | 11 | **room** |
| 63.62(54.22,99.38) | 9 | 63.78(49.18,104.33) | 9 | 64.39(57.99,94.44) | 9 | **Others** |
| 0.209 |  | 0.553 |  | 0.190 |  | **P value** |
|  |  |  |  |  |  | **Heating** |
| 66.84(39.66,179.26) | 100 | 67.44(45.42,177.12) | 100 | 67.93(6.83,181.4) | 102 | **Heater** |
| 63.62(49.66,143.21) | 35 | 63.78(38.1,159.09) | 35 | 64.39(40.36,127.33) | 35 | **Radiant** |
| 59.82(50.15,73.94) | 5 | 73.54(56.3,75.46) | 5 | 52.02(44.0,74.35) | 5 | **Others** |
| 0.404 |  | 0.422 |  | 0.280 |  | **P value** |
|  |  |  |  |  |  | **Cooling** |
| 64.35(39.66,143.21) | 119 | 65.25(38.1,159.09) | 119 | 64.6(6.83,128.23) | 121 | **cooler** |
| 71.97(54.99,179.26) | 21 | 71.3(54.99,177.12) | 21 | 68.77(52.02,181.4) | 21 | **cooler and Others** |
| 0.021 |  | 0.027 |  | 0.064 |  | **P value** |
|  |  |  |  |  |  | **Ventilation** |
| 74.84(54.89,123.35) | 6 | 71.3(46.23,1306) | 6 | 73.79(63.56,116.09) | 7 | **no Ventilation** |
| 64.63(39.66,143.21) | 101 | 66.19(38.1,159.09) | 101 | 64.98(6.83,127.33) | 101 | **Natural ventilation** |
| 68.01(50.34,179.26) | 33 | 64.99(49.67,177.12) | 33 | 65.19(43.72,181.4) | 34 | **Fan and hood** |
| 0.189 |  | 0.698 |  | 0.074 |  | **P value** |
|  |  |  |  |  |  | **Window tyPe** |
| 65.33(39.66,179.26) | 122 | 65.37(38.1,177.12) | 122 | 64.81(6.83,181.4) | 124 | **Normal** |
| 67.45(50.34,143.21) | 18 | 67.83(49.67,159.09) | 18 | 67.89(43.72,127.33) | 18 | **Two shells** |
| 0.357 |  | 0.440 |  | 0.433 |  | **P value** |
|  |  |  |  |  |  | **Kitchen tyPe** |
| 64.35(45.42,179.26) | 102 | 65.25(38.1,177.12) | 102 | 64.77(39.69,181.4) | 104 | **oPen** |
| 68.09(39.66,115.6) | 38 | 69.44(45.42,120.73) | 38 | 68.26(6.83,11.02) | 38 | **No oPen** |
| 0.538 |  | 0.968 |  | 0.303 |  | **P value** |
|  |  |  |  |  |  | **Kitchen ventilation** |
| 75.8(61.01,146.04) | 10 | 77.04(54.99,159.23) | 10 | 74.67(57.23,132.84) | 10 | **no Ventilation** |
| 65.33(47.08,179.26) | 35 | 63.09(45.42,177.12) | 35 | 64.81(45.59,181.4) | 35 | **Natural ventilation** |
| 64.28(39.66,143.21) | 93 | 65.25(38.1,159.09) | 93 | 63.81(6.83,128.23) | 95 | **Fan and hood** |
| 76.88(50.34,103.43) | 2 | 85.62(56.95,114.29) | 2 | 68.15(43072,92.57) | 2 | **Others** |
| 0.053 |  | 0.062 |  | 0.081 |  | **P value** |
|  |  |  |  |  |  | **Number of floors** |
| 67.29(45.42,179.26) | 24 | 70.11(48.56,177.12) | 24 | 65.27(40.52,181.4) | 24 | **Underground** |
| 65.33(39.66,143.21) | 100 | 65.17(45.42,159.09) | 100 | 66(6.83,127.33) | 102 | **first floor or ground level** |
| 61.96(49.66,127.98) | 16 | 66.06(38.1,127.74) | 16 | 63.11(40.36,128.23) | 16 | **More than two floors** |
| 0.406 |  | 0.650 |  | 0.534 |  | **P value** |
|  |  |  |  |  |  | **Building age** |
| 64.27(39.66,179.26) | 96 | 63.98(38.1,177.12) | 96 | 63.5(6.83,181.4) | 97 | **Under 20 years old** |
| 69.12(48.11,115.6) | 44 | 71.54(45.42,120.73) | 44 | 69.36(39.69,11.02) | 45 | **Over 20 years old** |
| 0.038 |  | 0.108 |  | 0.045 |  | **P value** |
|  |  |  |  |  |  | **building tyPe** |
| 62.91(49.66,143.21) | 24 | 63.03(38.10,159.09) | 24 | 63.53(44.00,127.33) | 26 | **the aPartment** |
| 66.77(39.66,179.26) | 115 | 66.23(45.42,177.12) | 115 | 67.46(6.83,181.40) | 115 | **Villa** |
| 0.210 |  | 0.391 |  | 0.253 |  | **P value** |

Table S5: Relationship between MS and building materials

| Chi-square | **Number(Percent)**  **Without MS** | **Number(Percent)**  **MS** | **Building characteristics** |
| --- | --- | --- | --- |
| 0.433 |  |  | **Foundation** |
|  | 43(43.0%) | 15(33.3%) | **Concrete** |
|  | 52(52.0%) | 26(57.8%) | **The brick** |
|  | 5(5.0%) | 4(8.9%) | **Others** |
| 0.015 |  |  | **structure** |
|  | 38(38.0%) | 12(26.7%) | **Concrete** |
|  | 36(36.0%) | 10(22.2%) | **Metal** |
|  | 11(11.0%) | 6(13.3%) | **reinforced concrete** |
|  | 15(15.0%) | 17(37.8%) | **Others** |
| 0.010 |  |  | **Materials used in the facade** |
|  | 14(14.3%) | 14(32.6%) | **Rock** |
|  | 60(61.2%) | 19(44.2%) | **Brick clay** |
|  | 12(12.2%) | 1(2.3%) | **ceramic** |
|  | 12(12.2%) | 9(20.9%) | **Others** |
| 0.021 |  |  | **Floor Covering** |
|  | 50(50.0%) | 32(71.1%) | **Ceramic and Tile** |
|  | 41(41.0%) | 13(28.9%) | **Mosaic** |
|  | 9(9.0%) | 0(0.0%) | **Others** |
| 0.283 |  |  | **Wall covering** |
|  | 14(14.0%) | 11(25.0%) | **Ceramic and tile** |
|  | 12(12.0%) | 8(18.2%) | **Plaster and Rock** |
|  | 25(25.0%) | 11(25.0%) | **Plaster and Ceramic** |
|  | 45(45.0%) | 13(29.5%) | **Plaster and Color** |
|  | 4(4.0%) | 1(2.3%) | **Others** |
| 1.00 |  |  | **Most used sPace** |
|  | 86(86.0%) | 39(86.7%) | **living room** |
|  | 8(8.0%) | 3(6.7%) | **room** |
|  | 6(6.0%) | 3(6.7%) | **Others** |
| 0.327 |  |  | **Heating** |
|  | 74(74.7%) | 30(68.2%) | **Heater** |
|  | 22(22.2%) | 14(31.8%) | **Radiant** |
|  | 3(3.0%) | 0(0.0%) | **Others** |
| 0.157 |  |  | **Cooling** |
|  | 82(82.0%) | 41(91.1%) | **air conditioner** |
|  | 18(18.0%) | 4(8.9%) | **air conditioner and Others** |
| 0.001 |  |  | **Ventilation** |
|  | 0(0.0%) | 7(15.6%) | **no Ventilation** |
|  | 75(75.0%) | 28(62.2%) | **Natural ventilation** |
|  | 25(25.0%) | 10(22.2%) | **Fan and hood** |
| 0.557 |  |  | **Window tyPe** |
|  | 88(88.0%) | 38(86.9%) | **Normal** |
|  | 12(12.0%) | 7(15.6%) | **Two shells** |
| 0.967 |  |  | **Kitchen tyPe** |
|  | 73(73.0%) | 33(73.3%) | **oPen** |
|  | 27(27.0%) | 12(26.7%) | **No oPen** |
| 0.448 |  |  | **Kitchen ventilation** |
|  | 5(5.0%) | 5(11.1%) | **no Ventilation** |
|  | 26(26.0%) | 9(20.0%) | **Natural ventilation** |
|  | 67(67.0%) | 31(68.9%) | **Fan and hood** |
|  | 2(2.0%) | 0(0.0%) | **Others** |
| 0.044 |  |  | **Number of floors** |
|  | 13(13.0%) | 11(24.4%) | **Underground** |
|  | 78(78.0%) | 26(57.8%) | **first floor** |
|  | 9(9.0%) | 8(17.8%) | **More than two floors** |
| 0.780 |  |  | **Building age** |
|  | 69(69.0%) | 30(66.7%) | **Under 20 years old** |
|  | 31(31.0%) | 15(33.3%) | **20 years uP** |
| 0.082 |  |  | **building tyPe** |
|  | 15(15.0%) | 12(27.3%) | **the aPartment** |
|  | 85(85.0%) | 32(72.7%) | **Villa** |

Table S6: Mean intake of food groups with MS and non-MS

|  | Case | Control | P. value |
| --- | --- | --- | --- |
| Processed meat (g/day)^d^ |  |  |  |
| Model I | 14.27±2.72^c^ | 1.33±1.82 | <0.001 |
| Model II^a^ | 13.56±2.71 | 1.52±1.80 | <0.001 |
| Model III^b^ | 14.21±3.49 | 1.50±2.20 | 0.004 |
| Organ meats (g/day) |  |  |  |
| Model I | 9.85±2.68 | 5.66±1.80 | 0.196 |
| Model II | 9.96±2.70 | 5.61±1.77 | 0.188 |
| Model III | 12.57±3.78 | 5.59±2.39 | 0.136 |
| Fish (g/day) |  |  |  |
| Model I | 9.84±2.40 | 11.87±1.61 | 0.484 |
| Model II | 10.17±2.42 | 11.72±1.60 | 0.599 |
| Model III | 11.11±3.33 | 12.64±2.11 | 0.709 |
| Poultry (g/day) |  |  |  |
| Model I | 61.32±13.16 | 59.27±8.83 | 0.897 |
| Model II | 53.98±12.09 | 62.58±8.00 | 0.560 |
| Model III | 61.06±13.35 | 57.40±8.45 | 0.824 |
| Eggs (g/day) |  |  |  |
| Model I | 28.74±3.16 | 22.53±2.12 | 0.105 |
| Model II | 28.09±3.25 | 22.82±2.15 | 0.185 |
| Model III | 31.48±4.24 | 22.71±2.69 | 0.096 |
| Butter (g/day) |  |  |  |
| Model I | 3.85±0.64 | 2.30±0.43 | 0.045 |
| Model II | 3.70±0.66 | 2.37±0.43 | 0.095 |
| Model III | 3.59±0.86 | 2.67±0.55 | 0.390 |
| Margarine (g/day) |  |  |  |
| Model I | 1.30±0.40 | 0.61±0.27 | 0.148 |
| Model II | 1.28±0.41 | 0.62±0.27 | 0.183 |
| Model III | 1.43±0.59 | 0.76±0.37 | 0.355 |
| Low-fat dairy Product (g/day) |  |  |  |
| Model I | 78.75±20.35 | 83.37±13.65 | 0.850 |
| Model II | 76.30±19.90 | 84.47±13.17 | 0.736 |
| Model III | 95.14±27.38 | 81.26±17.32 | 0.681 |
| High-fat dairy Product (g/day) |  |  |  |
| Model I | 164.68±37.97 | 189.11±25.47 | 0.594 |
| Model II | 137.30±34.85 | 201.43±23.06 | 0.133 |
| Model III | 138.97±46.75 | 199.10±29.58 | 0.298 |
| Tea (g/day) |  |  |  |
| Model I | 357.73±54.81 | 404.49±36.77 | 0.480 |
| Model II | 357.73±54.81 | 404.49±36.77 | 0.480 |
| Model III | 402.35±73.86 | 426.62±47.04 | 0.789 |
| Coffee (g/day) |  |  |  |
| Model I | 6.95±1.89 | 4.91±1.27 | 0.373 |
| Model II | 5.90±1.90 | 5.38±1.26 | 0.823 |
| Model III | 4.13±2.16 | 5.77±1.36 | 0.539 |
| Fruit (g/day) |  |  |  |
| Model I | 880.79±140.38 | 846.35±94.17 | 0.839 |
| Model II | 788.22±119.04 | 888.01±78.78 | 0.492 |
| Model III | 779.47±139.74 | 896.73±88.50 | 0.497 |
| Fruit juice (g/day) |  |  |  |
| Model I | 128.51±24.76 | 79.37±16.61 | 0.102 |
| Model II | 112.37±22.32 | 86.63±14.77 | 0.345 |
| Model III | 82.713±27.30 | 86.21±17.28 | 0.917 |
| Tomatoes (g/day) |  |  |  |
| Model I | 63.93±21.53 | 105.16±14.44 | 0.114 |
| Model II | 54.75±20.98 | 109.29±13.88 | 0.034 |
| Model III | 49.00±27.76 | 106.13±17.56 | 0.098 |
| Vegetables (g/day) |  |  |  |
| Model I | 238.41±33.00 | 208.77±22.14 | 0.457 |
| Model II | 224.97±28.47 | 214.81±18.84 | 0.770 |
| Model III | 228.42±32.79 | 204.50±20.77 | 0.554 |
| Garlic (g/day) |  |  |  |
| Model I | 0.59±0.23 | 0.86±0.15 | 0.323 |
| Model II | 0.46±0.22 | 0.92±0.15 | 0.090 |
| Model III | 0.62±0.25 | 0.70±0.16 | 0.786 |
| Potatoes (g/day) |  |  |  |
| Model I | 15.57±4.72 | 27.26±3.16 | 0.041 |
| Model II | 14.13±4.83 | 27.91±3.19 | 0.020 |
| Model III | 12.55±3.90 | 24.83±2.47 | 0.012 |
| Whole grain (g/day) |  |  |  |
| Model I | 60.41±12.78 | 72.50±8.57 | 0.433 |
| Model II | 54.76±12.44 | 75.05±8.23 | 0.182 |
| Model III | 66.33±16.34 | 75.75±10.34 | 0.640 |
| Refrains grain (g/day) |  |  |  |
| Model I | 269.44±30.74 | 253.82±20.62 | 0.674 |
| Model II | 247.01±28.14 | 263.91±18.62 | 0.623 |
| Model III | 225.91±29.25 | 262.67±18.51 | 0.310 |
| Snakes (g/day) |  |  |  |
| Model I | 4.80±2.09 | 7.37±0.40 | 0.310 |
| Model II | 4.34±2.16 | 7.57±1.43 | 0.221 |
| Model III | 3.93±3.10 | 8.35±1.96 | 0.249 |
| Dried fruit (g/day) |  |  |  |
| Model I | 10.79±2.37 | 10.50±1.59 | 0.918 |
| Model II | 10.83±2.43 | 10.48±1.61 | 0.906 |
| Model III | 11.59±3.19 | 11.18±2.02 | 0.918 |
| Mayonnaise (g/day) |  |  |  |
| Model I | 3.71±0.89 | 1.99±0.60 | 0.110 |
| Model II | 3.45±0.88 | 2.11±0.58 | 0.208 |
| Model III | 2.44±0.84 | 2.16±0.53 | 0.782 |
| Nuts (g/day) |  |  |  |
| Model I | 19.10±2.61 | 16.77±1.75 | 0.458 |
| Model II | 16.92±2.43 | 17.75±1.61 | 0.778 |
| Model III | 16.70±3.17 | 18.93±2.00 | 0.568 |
| Olive (g/day) |  |  |  |
| Model I | 6.65±1.60 | 4.21±1.07 | 0.207 |
| Model II | 6.20±1.62 | 4.41±1.08 | 0.366 |
| Model III | 5.63±2.28 | 5.16±1.44 | 0.868 |
| confectioneries (g/day) |  |  |  |
| Model I | 64.82±7.20 | 40.97±4.83 | 0.007 |
| Model II | 61.47±7.11 | 42.48±.70 | 0.030 |
| Model III | 68.32±8.69 | 38.12±5.50 | 0.006 |
| Hydrogenated fats (g/day) |  |  |  |
| Model I | 9.98±1.50 | 2.51±1.00 | <0.001 |
| Model II | 9.51±1.50 | 2.72±0.99 | <0.001 |
| Model III | 7.92±1.90 | 2.18±1.20 | 0.015 |
| Vegetable oil (g/day) |  |  |  |
| Model I | 8.55±1.75 | 7.65±1.17 | 0.667 |
| Model II | 7.68±1.77 | 8.04±1.17 | 0.865 |
| Model III | 7.99±1.94 | 7.70±1.23 | 0.905 |
| Sugars (g/day) |  |  |  |
| Model I | 32.71±4.76 | 26.28±3.20 | 0.263 |
| Model II | 31.82±4.46 | 26.68±2.95 | 0.344 |
| Model III | 35.28±5.2 | 25.15±3.34 | 0.122 |
| Condiment (g/day) |  |  |  |
| Model I | 28.60±3.80 | 23.99±2.55 | 0.316 |
| Model II | 26.42±3.63 | 24.97±2.40 | 0.742 |
| Model III | 29.32±4.85 | 26.41±3.07 | 0.627 |
| Soft drink (g/day) |  |  |  |
| Model I | 83.30±11.69 | 28.80±7.84 | <0.001 |
| Model II | 78.93±11.28 | 30.76±7.47 | 0.001 |
| Model III | 60.40±12.71 | 28.79±8.04 | 0.046 |
| Yogurt drink (g/day) |  |  |  |
| Model I | 60.78±22.30 | 88.02±14.96 | 0.312 |
| Model II | 53.61±22.42 | 91.25±14.84 | 0.170 |
| Model III | 43.45±30.76 | 100.17±19.47 | 0.137 |
| Broth (g/day) |  |  |  |
| Model I | 41.73±14.10 | 45.21±9.46 | 0.838 |
| Model II | 43.42±14.50 | 44.45±9.60 | 0.953 |
| Model III | 49.27±20.15 | 56.28±12.75 | 0.778 |
| Salt (g/day) |  |  |  |
| Model I | 6.67±1.18 | 6.47±0.79 | 0.887 |
| Model II | 6.21±1.20 | 6.68±.80 | 0.753 |
| Model III | 6.21±1.42 | 6.65±0.70 | 0.800 |
| Pickles (g/day) |  |  |  |
| Model I | 13.34±12.09 | 28.31±8.11 | 0.306 |
| Model II | 7.42±12.32 | 30.97±8.15 | 0.119 |
| Model III | 11.26±4.15 | 21.13±2.63 | 0.056 |
| Legumes (g/day) |  |  |  |
| Model I | 32.68±8.77 | 44.08±5.88 | 0.282 |
| Model II | 28.29±8.16 | 46.05±5.40 | 0.076 |
| Model III | 35.74±11.07 | 41.79±7.00 | 0.658 |
| Red meats (g/day) |  |  |  |
| Model I | 85.77±8.30 | 62.17±5.57 | 0.020 |
| Model II | 81.10±7.96 | 64.27±5.26 | 0.084 |
| Model III | 83.23±9.58 | 63.42±6.06 | 0.096 |
